# Optimal Treatment Selection for Cesarean Scar Pregnancy: Using a Predictive Score of Intraoperative Blood Loss: A Prospective Cohort Study

**DOI:** 10.1002/hsr2.72984

**Published:** 2026-08-04

**Authors:** Yunhui Tang, Man Li, Cuifeng Qian, Lei Zhu, Yang Gao, Hua Feng, Fuqin Zhang, Xiaoying Yao, Qi Chen

**Affiliations:** ^1^ Department of Family Planning, The Hospital of Obstetrics and Gynecology Fudan University Shanghai China; ^2^ Department of Ultrasound, The Hospital of Obstetrics and Gynecology Fudan University Shanghai China; ^3^ Department of Hysteroscopic diagnosis and treatment, The Hospital of Obstetrics and Gynecology Fudan University Shanghai China; ^4^ Department of Obstetrics and Gynecology The University of Auckland Auckland New Zealand

**Keywords:** cesarean scar pregnancy, intraoperative blood loss, quantitative risk‐scoring system, suction evacuation, uterine artery embolization

## Abstract

**Background and Aims:**

The significant risk associated with treatment for cesarean scar pregnancy (CSP) is the potential for severe hemorrhage at termination, resulting in controversy over optimal treatment and no international standards. Our previous study proposed that estimating intraoperative blood loss from ultrasound findings, called quantitative risk‐scoring (QRS), may help select the optimal treatment. In this study, we analyzed the success rate in selecting the optimal treatment for CSP based on QRS.

**Methods:**

A total of 318 CSP cases were included, and the initial treatment, including suction evacuation or uterine artery embolisation (UAE), was selected based on intraoperative blood loss estimates. Failure and intraoperative blood loss were measured.

**Results:**

The mean intraoperative blood loss in CSP cases with QRS < 3 who underwent suction evacuation were not statistically different from CSP cases with UAE. In CSP cases with QRS ≥ 3, there were 14 (10%) or 6 (8%) unsuccessful cases with suction evacuation or UAE. The mean intraoperative blood loss after excluding failure was not statistically different between the two groups. Additionally, type 2 CSP cases with QRS ≥ 3 and UAE had lower intraoperative blood loss than those who initially underwent hysteroscopy or laparoscopy, with 5 (8%) cases being unsuccessful. Our study suggested that suction evacuation is safe, with a higher success rate and minimal blood loss for cases with QRS < 3. Similarly, the UAE is safe with minimal blood loss for CSP cases with QRS ≥ 3, although the unsuccessful rate was slightly higher.

**Conclusions:**

Our study suggests that QRS can be a tool for selecting initial treatment for CSP in clinical practice.

## Summary

Intraoperative blood loss during surgical treatment for CSP is a risk factor, and we previously developed a QRS to estimate it. In this prospective study, we validated that QRS can serve as an indicator for selecting initial surgical treatment.

**Abbreviations**
BMI: body mass index
CSP: cesarean scar pregnancy
QRS: quantitative risk‐scoring
SD: standard deviation
UAE: uterine artery embolization
β‐hCG: beta‐human chorionic gonadotropin

## 1 Introduction

Cesarean scar pregnancy (CSP) is defined as the implantation of an embryo in uterine scar tissue from a previous cesarean section. The incidence of CSP has significantly increased due to the global increase in cesarean section rates. The higher incidence of CSP has been mainly seen in China since the end of the One‐Child Policy in 2015. This is because China has the highest cesarean section rate worldwide [1]. Continuing with a CSP significantly increases the intrinsic risk of uterine rupture, placenta accreta spectrum [2, 3], severe hemorrhage, and even hysterectomy, which leads to life‐threatening [4, 5, 6]. Early termination of CSP is generally recommended to minimize complications [7, 8]. Despite numerous treatment options that have been suggested, there is no standard optimal treatment or a single best option for CSP management due to a lack of evidence‐based guidelines [9, 10]. Recent studies conducted in the United Kingdom have shown that surgery has a high success rate (92%–96%) with a low complication rate and short post‐treatment follow‐up for CSP treatment [8, 11]. However, a uterine arteriovenous malformation after suction evacuation was recently reported [12].

The nature of CSP can lead to an abnormally adherent placenta, which could result in severe intraoperative blood loss or even a hysterectomy at the time of operation or delivery [6], which is a significant clinical challenge. However, the amount of blood loss can vary depending on the severity of the condition and the individual case factors, such as the extent of blood vessel invasion. Increasing evidence suggests that the size (diameter) of the gestational sac and gestational age are likely risk factors for severe blood loss during CSP treatment [13], as well as the subtypes of CSP [14]. Additionally, serum levels of beta‐human chorionic gonadotropin (β‐hCG) have also been reported as risk factors for severe intraoperative blood loss [15, 16]. Surgical suction evacuation with or without uterine artery embolisation (UAE) under ultrasound guidance is suggested to be safe with minimal intraoperative blood loss [17, 18, 19]. However, there is currently a lack of studies that pre‐assess the risk of intraoperative blood loss in CSP surgical treatment to select an optimal treatment. Lack of pre‐assessing the risk of intraoperative blood loss consequently resulted in an overuse of UAE in many cases, regardless of the extent of embryo invasiveness into the uterine wall, specifically in China. We recently proposed a quantitative risk‐scoring (QRS) method to estimate intraoperative blood loss in CSP based on gestational age and ultrasound‐detected blood flow around the gestational sac [20]. Our pilot study indicated a lower risk of intraoperative blood loss when the QRS is less than 3 [20].

Traditional CSP classification outlines two subtypes (type 1 and type 2) based on the implantation site of the gestational sac and the thickness of the remaining myometrium [10, 21, 22]. More recently, a three‐subtype classification (type 1, type 2, and type 3) was proposed according to the surgical management strategy [23] Additionally, the newer Crossover Sign (COS) classification focuses on the relationship between the gestational sac and the previous scar to better predict the risk of placenta accreta spectrum and uterine rupture [24, 25].

To investigate the effect of using QRS in selecting an optimal treatment for CSP, we conducted a large, prospective, observational study to assess intraoperative blood loss and the number of failures in the selected initial treatment, accounting for traditional CSP subtypes. The primary objective of this study is to investigate whether QRS can serve as an indicator or predictor of the selection of initial surgical treatment.

## 2 Methods

### 2.1 Study Cohort

The Hospital of Obstetrics and Gynecology, Fudan University, Shanghai, China, is one of the largest Obstetrics and Gynecology specialized hospitals in the country, ranked among the top three nationally. More than 150 women who are primarily diagnosed with CSP by a transvaginal ultrasound annually admitted to our Family Planning clinic. The procedure and management protocol in our hospital are based on the gestational age of the sac and hemodynamic stability, and currently QRS. The treatment options, either surgical suction evacuation with or without uterine artery UAE under ultrasound guidance, were then discussed with women. The gynecologists for performance treatment and sonologists are senior consultants. Women are followed up starting one or 2 weeks after the operation, until the β‐hCG levels reach undetectable levels.

**Figure 1:**
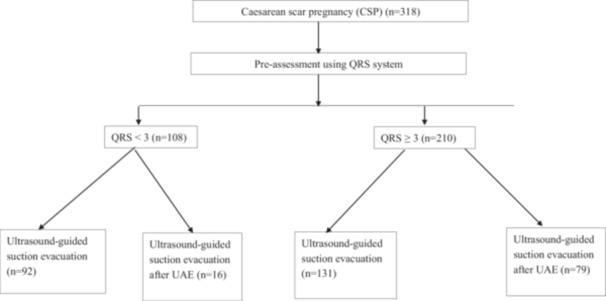
Study flow chart.

318 women diagnosed with CSP were included in this prospective study who attended the Hospital of Obstetrics and Gynecology at Fudan University from December 2019 to July 2023. The diagnosis of CSP was based on the transvaginal ultrasound findings, including the presence of a gestational sac in the area of the scar tissue, with or without fetal cardiac activity on Doppler flow ultrasonogram (Mindray, China), in addition to a history of prior cesarean section and positive pregnancy test [21, 26]. Two individual gynecologic ultrasonography physicians confirmed the diagnosis. All patients were first assessed by the QRS to estimate intraoperative blood loss after diagnosis, and the initial treatment was then decided based on the QRS and the subtypes of CSP (Figure 1). Details of the QRS are listed in Table 1. The initial treatment included suction evacuation (curettage) with ultrasound guidance or suction evacuation with ultrasound guidance after uterine artery embolisation (UAE) using a 5‐F angiographic catheter. All these cases were elective, and none of our CSP patients received systemic methotrexate treatment based on our hospital protocol.

**Table 1:** Quantitative risk scoring (QRS) system for the prediction of intraoperative blood loss (cited from reference [20]).

Variable	Score
Vascularity around the gestational sac*	
No obvious blood flow	0
Sparse or sporadic blood flow	1
The vessel looks like a short‐columnar pattern	2
The vessel looks cord‐like pattern	3
Massive blood flow or arteriovenous fistula	4
Pregnancy duration	
≤ 40 days	0
41–50 days	1
> 50 days	2

* As demonstrated on a color Doppler ultrasonography.

Data on maternal age, parity, and gravidity, gestational age or size of sac of current CSP, the serum levels of β‐hCG, subtypes of CSP, interval from the previous cesarean section to current CSP, initial surgical treatment, ultrasound findings, amount of intraoperative blood loss, blood transfer, operation time, and the failure of the initial treatment were collected. In addition, follow‐up data, including the days to return to the menstrual cycle and ultrasound findings, were collected.

Several studies suggest that using hysteroscopy, laparoscopy, or a combination of the two for treating type 2 CSP has a higher success rate [27]. We then collected the same data from 17 type 2 cases who initially received surgical treatment with hysteroscopy or laparoscopy, and the QRS were retrospectively assessed as greater than 3 during the same period of the study. To ensure consistence with other study cohorts, the retrospective QRS evaluation of the 17 surgical cases was performed using objective, archived pre‐operative ultrasound criteria, independently of the clinical outcome data. We also compared the outcomes between these cases and type 2 CSP cases with QRS ≥ 3 who received suction evacuation after UAE (*n* = 62). To evaluate for potential selection bias between the prospective UAE cohort and the retrospective surgical cohort, baseline maternal age, gestational age, and pre‐treatment serum β‐hCG levels were compared using Mann‐Whitney U test.

**Figure 2:**
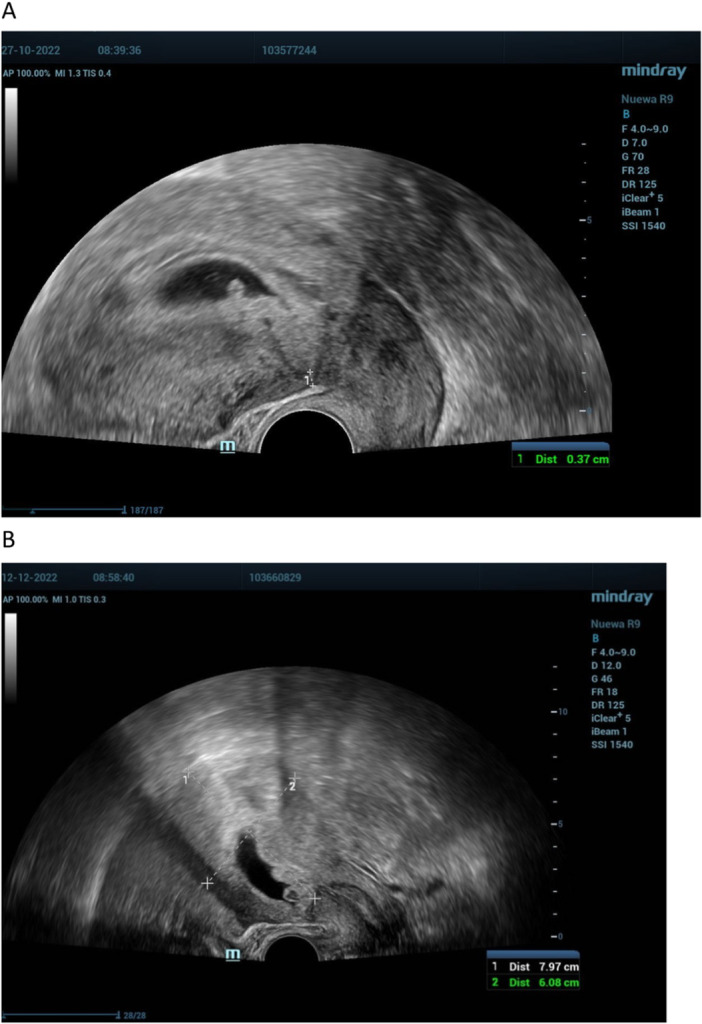
Ultrasound images showing type 1 (A) and type 2 CSP (B).

Intraoperative blood loss was estimated using an autologous cell salvage system. Blood was suctioned during the operation and filtered, while clots and tissues were also collected and centrifuged using a mesh strainer. The total amount of intraoperative blood loss was calculated as the sum of cumulative volume from both collection methods. Type 1 CSP is defined when the embryo is implanted in the myometrium and grows toward the cervico‐isthmic space or uterine cavity (Figure 2A). Type 2 CSP (Figure 2B) is defined when the embryo is implanted deeply within the scar tissue and penetrates the uterine wall, sometimes reaching the uterine blood vessels, as determined by ultrasound [10, 21, 22].

The failure of initial treatment was defined as when the initial treatment was unsuccessful, leaving residual tissues, or when intraoperative blood loss could not be controlled by the initial treatment; the additional option was then used. Severe intraoperative blood loss was defined as exceeding 100 ml during the initial surgical treatment.

### 2.2 Statistical Analysis

Data were presented as means and standard deviations (SD). The normality of data, including maternal age, body mass index (BMI), β‐hCG, and gestational age, was assessed using GraphPad Prism. Missing data, including β‐hCG and the interval between two pregnancies, were handled using complete‐case analysis (Listwise Deletion). Missing data was assumed to be missing completely at random. The missingness included in this study was well below the 5%–10% threshold, suggesting that further analysis did not introduce significant bias or compromise statistical power. T‐test (Mann‐Whitney test) or Fisher's exact test was performed for statistical analysis using GraphPad Prism (version 10.1.0). Statistical difference was considered when *p* < 0.05.

## 3 Results

The clinical parameters of women in this prospective study are summarized in Table 2. The mean maternal age at diagnosis was 36 (±17) years. The mean gestational age was 50 (±11) days. 18 (5%) cases had at least one previous CSP. 138 (43%) cases were identified as type 1 CSP. The mean interval between the last cesarean section and the current CSP was 7.4 (±4.0) years. 108 (34%) cases were assessed with the QRS < 3, and 210 (66%) with the QRS ≥ 3. 223 (70%) cases initially received suction evacuation, and 95 (30%) received suction evacuation after UAE.

**Table 2:** Clinical information of study cohort (*n* = 318).

Maternal age (years, mean/SD)	36 ± 17
BMI (kg/m^2^, mean/SD)	22.6 ± 3.6
Gestational age of sac (days, mean/SD)	50 ± 11
β‐hCG before treatment (mIU/mL, mean/SD)*	103791 ± 702761
Number of previous CS (*n*, %)	
1	94 (61%)
2	113 (36%)
> 3	111 (3%)
The interval between the last CS and current CSP (years, mean/SD)*	7.4 ± 4.0
With a history of previous CSP (*n*, %)	16 (5%)
Subtypes of CSP (*n*, %)	
Type 1	138 (43%)
Type 2	180 (57%)
QRS group (*n*, %)	
< 3	108 (34%)
≥ 3	210 (66%)
Initial treatment	
Suction evacuation with ultrasound	223(70%)
Uterine artery embolization	95 (30%)
Hemoglobin (g/L, mean/SD)*	121 ± 13

Abbreviations: β‐hCG, beta‐human chorionic gonadotropin; BMI, body mass index; CS, cesarean section; CSP, cesarean scar pregnancy; QRS, quantitative risk scoring; SD, standard deviation.

* 19 data missing in β‐hCG and hemoglobin, and 1 data missing in the interval between the last CS and current CSP.

In cases with QRS < 3 (*n* = 108), 92 received suction evacuation. The mean intraoperative blood loss in these cases was 13 (±30) ml, which was not statistically different compared with cases who received suction evacuation after UAE (*n* = 16), with 11 ml intraoperative blood loss (*p* = 0.602). We further analyzed intraoperative blood loss by CSP subtype between the two treatments. There were 56 (58%) type 1 CSP and 40 (42%) type 2 CSP in groups with suction evacuation, while there were 4 (25%) type 1 CSP and 12 (75%) type 2 CSP in a group with suction evacuation after UAE.

There was no statistical difference in intraoperative blood loss between the two groups, regardless of CSP subtype (Table 3 *p* > 0.05). There was only one case with severe intraoperative blood loss (300 ml), in which the patient initially underwent suction evacuation, and no cases were unsuccessful with the initial treatment.

**Table 3:** The comparison of intraoperative blood loss in cases with QRS < 3 (*n* = 108).

	Suction evacuation (*n* = 92)	UAE (*n* = 16)	*p* value (Mann‐Whitney test)
Blood loss (ml, mean/SD)	13.7 ± 30	11.1 ± 7.2	*p* = 0.6023
Blood loss in type 1 CSP (ml, mean/SD)	16 ± 38	6.8 ± 4.0	*p* > 0.05
Blood loss in type 2 CSP (ml, mean/SD)	10 ± 8.3	12.5 ± 7.5	*p* = 0.147
β‐hCG before treatment (mIU/mL, mean/SD)*	32631 ± 53981	56605 ± 52181	*p* = 0.02

Abbreviations: β‐hCG, beta‐human chorionic gonadotropin; CSP, cesarean scar pregnancy; QRS, quantitative risk scoring; SD, standard deviation; UAE, uterine artery embolization.

* 2 or 2 Data missing in the suction evacuation group or the UAE group.

In cases with QRS ≥ 3 (*n* = 210), 131 (62%) initially received suction evacuation, and 79 (38%) received suction evacuation after UAE (Table 4). There were 14 (10%) or 6 (8%) unsuccessful cases in the suction evacuation group or in the UAE treatment group at the initial treatment. In all successful cases with initial treatment, the mean intraoperative blood loss during suction evacuation after UAE was 14 (±14) ml, which was not statistically different from that in the suction evacuation group (12.5 ± 12 ml).

**Table 4:** The comparison of intraoperative blood loss in cases with QRS ≥ 3 (*n* = 210).

	Suction evacuation (*n* = 131)	UAE (*n* = 79)	*p* value
Blood loss* (ml, mean/SD)	12.5 ± 12.6	14 ± 14.3	*p* = 0.1774 (Mann‐Whitney test)
Failure (*n*, %)	14 (10%)	6 (8%)	*p*= 0.6285 (Mann‐Whitney test)
Operation time (minutes, mean/SD)	15 ± 10	17 ± 22	*p* = 0.3324 (Mann‐Whitney test)
Severe blood loss (*n*, %)	5 (4.0%)	4 (5.0%)	*p* = 0.7306 (Fisher's exact test)
β‐hCG before treatment (mIU/mL, mean/SD)**	168841 ± 1111519	93975 ± 85227	*p* = 0.0291 (Mann‐Whitney test)

Abbreviations: β‐hCG, beta‐human chorionic gonadotropin; QRS, quantitative risk scoring; SD, standard deviation; UAE, uterine artery embolization.

* Excluding the failed cases by the initial treatment.

** 14 or 1 data missing in the suction evacuation group or the UAE group.

Several studies suggested that using hysteroscopy, laparoscopy, or a combination of these for treating type 2 CSP has a higher success rate [27]. We then compared outcomes in type 2 CSP cases with QRS ≥ 3 who received suction evacuation after UAE (*n* = 62) with those who received hysteroscopy with or without laparoscopy and the QRS (≥ 3) were retrospectively assessed (*n* = 17). The baseline characteristics of the two groups are summarized in Table 5. There were no statistically significant differences in key baseline markers of bleeding risk, including gestational age at diagnosis (*p* = 0.2892), serum β‐hCG levels before treatment (*p* = 0.6511), and the individual ultrasound parameters comprising the QRS score. There were 5 (8%) unsuccessful cases in which suction evacuation was initially performed after the UAE. Excluding unsuccessful cases with initial treatment, the mean intraoperative blood loss in type 2 CSP cases with QRS ≥ 3 who received suction evacuation after UAE was 13 (±9) ml, which was significantly lower than that in cases with hysteroscopy with or without laparoscopy (178 ± 287 ml, *p* < 0.0001). One case had a hysterotomy in this group. The mean time to return to the menstrual cycle in cases who received suction evacuation after UAE was 33 (±10) days, significantly longer than in those who received hysteroscopy with or without laparoscopy (31 ± 8 days, *p* = 0.026).

**Table 5:** The comparison of the baseline characteristics between type 2 cases who received suction evacuation after UAE and the QRS were prospectively assessed and type 2 cases who initially received surgical treatment and the QRS were retrospectively assessed (Both groups with a QRS ≥ 3).

	Suction evacuation after UAE (*n* = 62)	Surgical treatment (*n* = 17)	*p* value (Mann‐Whitney test)
Maternal age (years, mean/SD)	35 ± 4.0	35 ± 3.7	*p* = 0.6841
Gestational age of sac (days, mean/SD)	52 ± 10	57 ± 13	*p* = 0.2892
BMI (kg/m^2^, mean/SD)	23 ± 3.4	22 ± 3.6	*p* = 0.1097
β‐hCG before treatment (mIU/mL, mean/SD)	78692 ± 69908	87079 ± 76342	*p* = 0.6511

## 4 Discussion

In this prospective and observational study, we investigated whether optimal treatment selection for CSP can be based on estimating intraoperative blood loss assessed by QRS, with primary focus on intraoperative blood loss and initial treatment failure as key outcome measures. Our data showed minimal intraoperative blood loss in successful cases with QRS < 3 who initially received suction evacuation. Conversely, we found minimal intraoperative blood loss in successful cases with QRS ≥ 3 who initially received suction evacuation after UAE, along with 13% failure.

Notably, most existing studies on CSP treatment had small sample sizes, underscoring the need for more evidence‐based guidelines. Surgical approaches in clinical practice, including ultrasound‐guided suction evacuation or suction evacuation after UAE and hysteroscopy in conjunction with or without laparoscopy, are commonly used. To avoid severe intraoperative blood loss, one of the serious challenges for CSP treatment, the options of surgical treatment are likely dependent on the experience of individuals. However, the effectiveness of the different options for CSP treatments is controversial [11, 28]. We have previously developed a QRS system to estimate intraoperative blood loss. In this study, we used our previously established QRS system to validate the selection of initial treatment for CSP.

In cases with QRS < 3, compared with cases with UAE as one of the options to reduce intraoperative blood loss, we found minimal intraoperative blood loss with suction evacuation, similar to that in cases that received suction evacuation after UAE. Intraoperative blood loss was higher in type 2 CSP, regardless of treatment option, in our current study, which agreed with a previous study [14]. However, this difference in intraoperative blood loss did not reach statistical significance. Serum levels of β‐hCG have also been reported as potential risk factors for severe intraoperative blood loss [15, 16]. However, a recent study found that higher serum β‐hCG levels were not associated with intraoperative blood loss [14]. Our current study showed significantly higher β‐hCG levels in cases that underwent suction evacuation after UAE. Still, intraoperative blood loss in this group was similar to that in suction evacuation cases. Taken together, our data suggests that the initial suction evacuation treatment is safe and has a higher success rate in cases with QRS < 3, regardless of CSP subtypes.

Our study showed an overall unsuccessful rate of 18% in cases with QRS ≥ 3 following initial treatments. This rate of failure did not differ significantly between the two treatments. This unsuccessful rate did not differ between type 1 and type 2 CSP with suction evacuation. In contrast, a higher unsuccessful rate was observed in type 2 CSP (82%) compared with type 1 CSP (18%) that underwent UAE. Interestingly, intraoperative blood loss in successful cases receiving initial treatments was minimal between the two initial treatments, after excluding unsuccessful cases. No difference in severe intraoperative blood loss was observed between the two initial treatments. While the UAE is not uncommonly used to control intraoperative blood loss for CSP treatment, especially in type 2 CSP [29], a recent study reported similar estimated intraoperative blood loss between cases with UAE and without UAE [14]. Additionally, there is currently no data regarding the outcomes of subsequent pregnancy in cases with UAE, resulting in a lack of international guidelines recommending UAE as an optimal option, especially for women who want future childbearing [30]. Our data suggest that, even in cases with QRS ≥ 3, suction evacuation may be safe, with minimal intraoperative blood loss, despite a relatively higher unsuccessful rate.

It is well known that type 2 CSP is associated with a higher risk of intraoperative blood loss than type 1 CSP. Therefore, several case series with relatively small sample sizes suggested that hysteroscopy, with or without laparoscopy, may achieve a higher success rate in controlling intraoperative blood loss in type 2 CSP [27]. A recent randomized clinical trial found that hysteroscopy was more effective than ultrasound‐guided suction evacuation [31]. However, this is not the case in our current study. When we compared intraoperative blood loss between type 2 CSP with QRS ≥ 3 who underwent suction evacuation after UAE and those who underwent hysteroscopy with or without laparoscopy, we found that intraoperative blood loss was significantly less in those who successfully underwent suction evacuation after UAE. At the same time, the rate of unsuccessfulness was considerably higher in this group. Because there were no statistically significant differences in key baseline markers of bleeding risk between the two groups (Table 5), the marked reduction in intraoperative blood loss in the UAE group (13 mL) is highly likely to reflect the true therapeutic effect of pre‐evacuation devascularization. The time to return to the menstrual cycle after initial suction evacuation following UAE in type 2 CSP with QRS ≥ 3 was significantly longer than in type 2 cases with QRS ≥ 3 after hysteroscopy with or without laparoscopy. However, the difference between the two groups was only 2 days (31 vs 33 days). Our data suggested that suction evacuation after UAE remains safe, with fewer intraoperative blood losses in type 2 CSP with higher QRS, and that this treatment did not cause adverse outcomes.

In our current study, the majority of women with CSP experienced an intraoperative blood loss of under 100 ml. The volume of intraoperative blood loss was substantially lower than the conventional definition of severe bleeding in general gynecological procedures, indicating a highly favorable outcome for CSP management by the treatment options we included in our study.

Our study has certain limitations that warrant consideration of our analysis. Our study was designed as a prospective and observational study, rather than a randomized controlled trial (RCT). This design can induce an inherent selection bias and limit our ability to draw causal conclusions. However, the main objective of this study was to validate the QRS we previously established [20], whether it correlates with the outcomes of CSP treatment selection. Additionally, RCT is not ethically feasible in our hospital. Finally, the QRS scores for type 2 cases who initially received surgical treatment with hysteroscopy or laparoscopy (*n* = 17) were retrospectively assessed. This may introduce inherent selection or information bias compared to the prospectively managed other cases, and the comparison of the type 2 CSP cases with QRS ≥ 3 is limited by the unequal sample sizes and the retrospective scoring of the surgical control group. However, there was no difference in the baseline characteristics between the two groups, suggesting the reduction of intraoperative blood loss in the UAE group could be the treatment effect.

## 5 Conclusions

Our current prospective and observational study indicates that in CSP cases with QRS < 3, suction evacuation alone is safe, with a higher success rate and minimal blood loss, regardless of subtypes. In CSP cases with QRS ≥ 3, although the unsuccessful rate was slightly higher in the suction evacuation groups compared to the suction evacuation after the UAE group, intraoperative blood loss was similar between the two groups. The optimal treatment selected in this study was based on the QRS system, and intraoperative blood loss in all successful cases with initial treatment was minimal and similar across optimal treatments. Our study may suggest that QRS can be a tool for selecting initial treatment for CSP in clinical practice.

## Author Contributions

**Yunhui Tang:** conceptualization, investigation, writing – original draft, data curation. **Man Li:** conceptualization, data curation, investigation. **Cuifeng Qian:** conceptualization, data curation. **Lei Zhu:** conceptualization, data curation. **Yang Gao:** conceptualization, data curation. **Hua Feng:** conceptualization. **Fuqin Zhang:** conceptualization, data curation. **Xiaoying Yao:** conceptualization, investigation. **Qi Chen:** conceptualization, investigation, writing – original draft, writing – review and editing.

## Disclosure

The lead author (Qi Chen) affirms that this article is an honest, accurate, and transparent account of the study being reported; that no important aspects of the study have been omitted; and that any discrepancies from the study as planned have been explained.

## Ethics Statement

This prospective study was approved by the Ethical Committee of The Hospital of Obstetrics and Gynecology, Fudan University, China (reference numbers: KYY2020‐185 and KYY 2019‐76). The informed consent form was collected from all participants. No information on identified participants was included in this manuscript. This study was performed in accordance with the Medical Association Declaration of Helsinki.

## Conflicts of Interest

All authors have no conflict of interest to report.

## Data Availability

The data that support the findings of this study are available on request from the corresponding author. The data are not publicly available due to privacy or ethical restrictions.
